# Round the Hospitals

**Published:** 1916-05-20

**Authors:** 


### ROUND THE HOSPITALS.

Some excellent people who have associated themselves with nursing, and aspire to leadership in the profession, have for years maintained an attitude which makes them notorious as agitators who are always tilting at those who are at present responsible for the education and training of nurses. The latest fad has been to start an agitation among the general body of nurses in favour of the view "that what members of the nursing profession require before they can attain to the highest standards is a sense of their own responsibility for their own profession." The object of this agitation is plainly stated to be "the maintenance and growth of a feeling amongst nurses that State registration should come before the organisation of a College of Nursing." We have just shown that the overwhelming feeling of Scotch nurses as voiced by Miss Pike is the exact contrary of the agitators' view, and we have abundant evidence to prove that the Scottish view is upheld by an overwhelming majority of trained nurses in England and Wales.

Another point which convicts the agitators of grave ignorance is the suggestion "that the College of Nursing now incorporated cannot be the product of hard thought and work and patience on the part of the nursing profession as a whole." If the College of Nursing has one ground in particular which entitles it to the respect and confidence of the great body of nurses throughout the United Kingdom, it is the fact that it is the product of hard thought and work and patience on the part of the real leaders of nursing opinion, who are in the closest touch with the great body of nurses. Every one of the leaders who are members of the Council of the College does practically represent the feelings, views, and wishes of an infinitely larger body of trained nurses than any other individuals or organisations can claim to voice. Each leader has to a greater or less extent for many years striven hard to develop in the large body of nurses who have come under her influence a sense of responsibility, and to equip them to exercise a sound judgment upon matters which affect the interests of their profession and their individual well-being. It is all fudge for people, some of whom are not even trained nurses, to try and bring experienced and certificated nurses to believe that the matrons who have taught them, and helped them, and know their requirements and views, "do not and cannot as a rule represent nurses' opinions," but merely the opinions of what these amateur critics describe as "the matrons' employers," whatever that may mean.

There are two further pretensions which certain agitators are advancing that do not rest upon knowledge or truth. One is that there exists a number of independent nurses' societies which are constituted on a democratic basis, and have been spontaneously formed by nurses themselves in defence of their profession and interests. We have made many inquiries with a view to discovering these societies, in the hope of obtaining copies of the democratic constitution which governs their working and procedure. All that we can discover is that certain hospitals, often at the instigation and under the presidency of the matron, have nurses' societies which include all the certificated nurses that have been trained at a particular school. These societies cannot, however, be in fact the political organisations which the agitators claim to exist, and we should welcome the production of a list of the societies referred to by the agitators, as they constitute a totally different type of organisation from the societies attached to hospitals which have training schools. The truth is, as Miss Gill pointed out in Scotland three weeks ago, every intelligent and independent nurse admits that the one thing the profession of nursing urgently requires at the moment is organisation, which the College of Nursing will speedily create on a democratic basis. Further, through its agency will rapidly come a perfected organisation of the whole nursing profession, which will be self-governed and rest upon the support of the whole body of trained nurses. Trained nurses in increasing numbers are every day realising that this is the actual position created by the founding of the College of Nursing, and they are well content with its first Council, for it includes representatives whom they know and have learned to trust, because of the excellent work they have done and are doing to help nurses of all grades to help themselves, and to secure them the State recognition which is their due.

The Association for the Promotion of the Registration of Nurses in Scotland now has a membership of 3,246, of which 338 new members joined in 1915. During the absence of Colonel Mackintosh and Mr. Antony Murray on war duties, Dr. Claud Ker and Miss Graham were elected joint hon. secretaries. At the annual meeting of the Association, Miss Pike, matron of Siberton Cottage Hospital, made a strong protest against an attempt made by three members to delay the Association's support of the College of Nursing on the ground that nurses should be made fully aware of its proposals and objects. Miss Pike stated that nurses were well informed on these matters by the nursing papers, and that they had a very good understanding of what the College of Nursing meant. This view was supported by all present, except the three objectors referred to. Miss Gill made an excellent point by insisting that the College would organise the profession, which every nurse admits is urgently required, and through the College there would be little difficulty in getting a Registration Bill passed. Seeing that the College, when established, is to be self-governed and democratic, it will rest with the nurses themselves as to whether they get registration or not.

At St. Bartholomew's Hospital money prizes are provided from a sum left by Mr. James Bentley, a former treasurer of the hospital, and Mr. Mathias Prime Lucas, a former Alderman of the

**MATRONS' AND SISTERS' DEPARTMENT—(*continued*).**

City of London, to be presented annually to sisters and nurses of the staff in recognition of their diligence and attention to patients. It is possible that the reason this plan has not been pursued more generally in the voluntary hospitals is the difficulty of awarding gifts of this kind with absolute impartiality to a few of a large staff of sisters and nurses working in a great hospital.

---

There can be no doubt that the remuneration of trained nurses in some hospitals and many institutions is not at present adequate, having regard to the increased cost of living due to the war, and it is a matter for great satisfaction that at the last meeting of the Council of the Queen Victoria's Jubilee Institute for Nurses, on the 10th inst., it was reported that, in reply to a letter sent by the Institute to the affiliated Associations with regard to the salaries of Queen's nurses, there was practical unanimity in agreeing with the Council that the salaries and allowances of the nurses are inadequate considering the increase in the cost of living. A considerable number of Associations have already increased the remuneration of their nurses, so as to bring it up to the new scale. When Queen Victoria's Jubilee Institute takes up this strong position on the point, there should be no doubt that every well-administered institution throughout the United Kingdom will at once take steps to increase the remuneration of their nurses by adopting the new scale referred to.

---

The Irishman was recovering from a sharp attack of illness, and had reached the stage when he had permission to get up for an hour. It was customary in the ward for the "sitting-up time" to be granted from tea- to bed-time. However, the Irishman held other views; and when the day nurses came on duty at an early hour they found him strolling around in a dressing-gown. On being remonstrated with, he expostulated against any curtailment of his activity. "Doctor said I could be up for an hour. He did not say any when, an' o' course I'll be after taking me hour reg'lar, and just makin' sure av it often and frequent."

---

*** It is our invariable practice to pay for all items of news which we may publish. The minimum payment is 5s. Paragraphs of about twenty lines in length—that is to say, of 150 words or less—are preferred. In this section during the war we propose to give 10s. to the correspondent who may send us in any week the best incident connected with hospital life and work. In this way we hope to afford to all practical aid and encouragement; while those who reciprocate will be able to render us invaluable service. Contributions should be addressed to The Editor, The Hospital, 28 and 29 Southampton Street, Strand, London, W.C., and, to be in time for the current week's issue, should reach him by the first post on Mondays.

